# Treatments and cancer: implications for radiologists

**DOI:** 10.3389/fimmu.2025.1564909

**Published:** 2025-04-16

**Authors:** Vincenza Granata, Roberta Fusco, Sergio Venanzio Setola, Assunta Borzacchiello, Francesca Della Sala, Ivano Rossi, Ludovica Ravo, Domenico Albano, Angelo Vanzulli, Antonella Petrillo, Francesco Izzo

**Affiliations:** ^1^ Division of Radiology, Istituto Nazionale Tumori IRCCS Fondazione Pascale – IRCCS di Napoli, Naples, Italy; ^2^ Institute of Polymers, Composites and Biomaterials, National Research Council (IPCB-CNR), Naples, Italy; ^3^ Division of Radiology, Università degli Studi di Napoli Federico II, Naples, Italy; ^4^ Diagnostic and Interventional Radiology Unit, IRCCS Istituto Ortopedico Galeazzi, Milan, Italy; ^5^ Dipartimento di Scienze Biomediche, Chirurgiche ed Odontoiatriche, Università degli Studi di Milano, Milano, Italy; ^6^ Department of Radiology, ASST Grande Ospedale Metropolitano Niguarda, Milan, Italy; ^7^ Department of Oncology and Hemato-Oncology, Università degli Studi di Milano, Milan, Italy; ^8^ Division of Epatobiliary Surgical Oncology, Istituto Nazionale Tumori IRCCS Fondazione Pascale—IRCCS di Napoli, Naples, Italy

**Keywords:** cancer treatment, radiology, imaging evaluation, liver cancer, personalized medicine

## Abstract

This review highlights the critical role of radiologists in personalized cancer treatment, focusing on the evaluation of treatment outcomes using imaging tools like Computed Tomography (CT), Magnetic Resonance Imaging (MRI), and Ultrasound. Radiologists assess the effectiveness and complications of therapies such as chemotherapy, immunotherapy, and ablative treatments. Understanding treatment mechanisms and consistent imaging protocols are essential for accurate evaluation, especially in managing complex cases like liver cancer. Collaboration between radiologists and oncologists is key to optimizing patient outcomes through precise imaging assessments.

## Introduction

In the era of personalized medicine, several therapeutic strategies are available for the management of cancer patients (1–3). Surgical resection is considered the curative treatment for resectable malignant tumors in patients considered eligible for surgery (4, 5). However, in locally advanced pathologies, the use of neoadjuvant therapy (e.g. radiotherapy combined with chemotherapy) eases the surgical procedure (less destructive treatments), having a considerable impact on the patient’s prognosis by reducing the risk of local recurrence after surgery (6–8). In patients who are not candidates for surgery, either due to the patient’s own conditions or the stage of the disease (metastatic), the possibility of combining multiple treatments, simultaneously or in different phases of disease management, has important implications for the patient’s survival and his quality of life (9–12). The main therapy for unresectable lesions is systemic therapy (cytotoxic chemotherapy, biological therapy, immunotherapy and their combinations) (13). Clinical trials completed in the last 5 years have demonstrated that adapting the treatment to the molecular and pathological characteristics of the tumor improves Overall Survival (OS) (14). In many neoplasms, especially those of the gastro-intestinal region, the genomic profile to detect somatic variants is fundamental for the identification of treatments that could be effective in a specific subset of patients. For 50% of patients with KRAS/NRAS/BRAF wild-type tumors, the combination of cetuximab and panitumumab (monoclonal antibodies against the epithelial growth factor receptor [EGFR]), with conventional chemotherapy, can improve median survival by 2-4 months compared to the use of the chemotherapy alone (15). In patients with microsatellite instability or mismatch repair deficiency, immunotherapy can be used as an up-front treatment (16). Furthermore, over the past decade, organ-directed treatments such as percutaneous ablation, intra-arterial embolic therapy, and targeted radiotherapy have proven to be extremely promising in the management of different tumors (17–22). In specific clinical conditions, such as small hepatocellular carcinoma (HCC <3 cm), percutaneous ablative therapies have become the first-line treatment (23), while intra-arterial embolic treatments are a cornerstone in patients with locally advanced disease (23). Radiotherapy may be the only treatment to be used in patients with rectal cancer in the initial stage of the disease (T2) or who are not eligible for chemotherapy treatment (24). In such a complex scenario, it seems clear that the role of the radiologist is crucial, just as knowledge of the treatment(s) a patient is undergoing is necessary.

### Treatment assessment

The treatment evaluation involves different phases, such as the evaluation of the technical success (for ablative treatments), the evaluation of the effectiveness of the treatment (e.g. dimensional reduction, devascularization and necrosis, fibrosis, etc.) and complications (25–27). The concept of “technical success” refers to the possibility of treating the target according to a standardized protocol (25). Complications are identified as any unexpected change in a procedural course, while adverse events are defined as any actual or potential treatment-related injury (25). Both should be evaluated according to the following classification systems: (a) the standards of the Common Terminology Criteria for Adverse Events, (b) the Clavien-Dindo classification, (c) the Society of Interventional Radiology classification, and (d) the Cardiovascular and Interventional Radiological Society of Europe Quality Assurance Document and Standards for Classification of Complications, and these complications should be characterized by severity and time of onset (e.g., during treatment, post-treatment, or delayed) (25). In the different phases of response evaluation, different diagnostic tools can be used, alone or in combination. Computed Tomography (CT) and Magnetic Resonance Imaging (MRI) are the most frequently used tools in the assessment of treatments and the choice of one rather than the other should consider the characteristics of the lesion (location, size, structure), the disease stage (local or widespread) and the type of treatment (radiotherapy, ablative therapies, immunotherapy, etc.) (28–32). CT should be preferred in patients with diffuse or oligometastatic disease, in lung lesions, and in the assessment of peritoneal carcinomatosis (33–36). MRI for brain, liver, pancreatic, and anorectal lesions (37–40). The ultrasound examination (US), without or with contrast medium (CEUS), is a tool often used as a support for problem solving during the pre- and post-treatment phases, as well as, for ablative treatments, during the procedure itself (41). Independently from the method used, it would be suitable to maintain consistency in the technique and study protocol used before a treatment and throughout the follow-up period.

## Tumors treatment effect: implication for imaging

### Conventional chemotherapy

**Figure 1:**
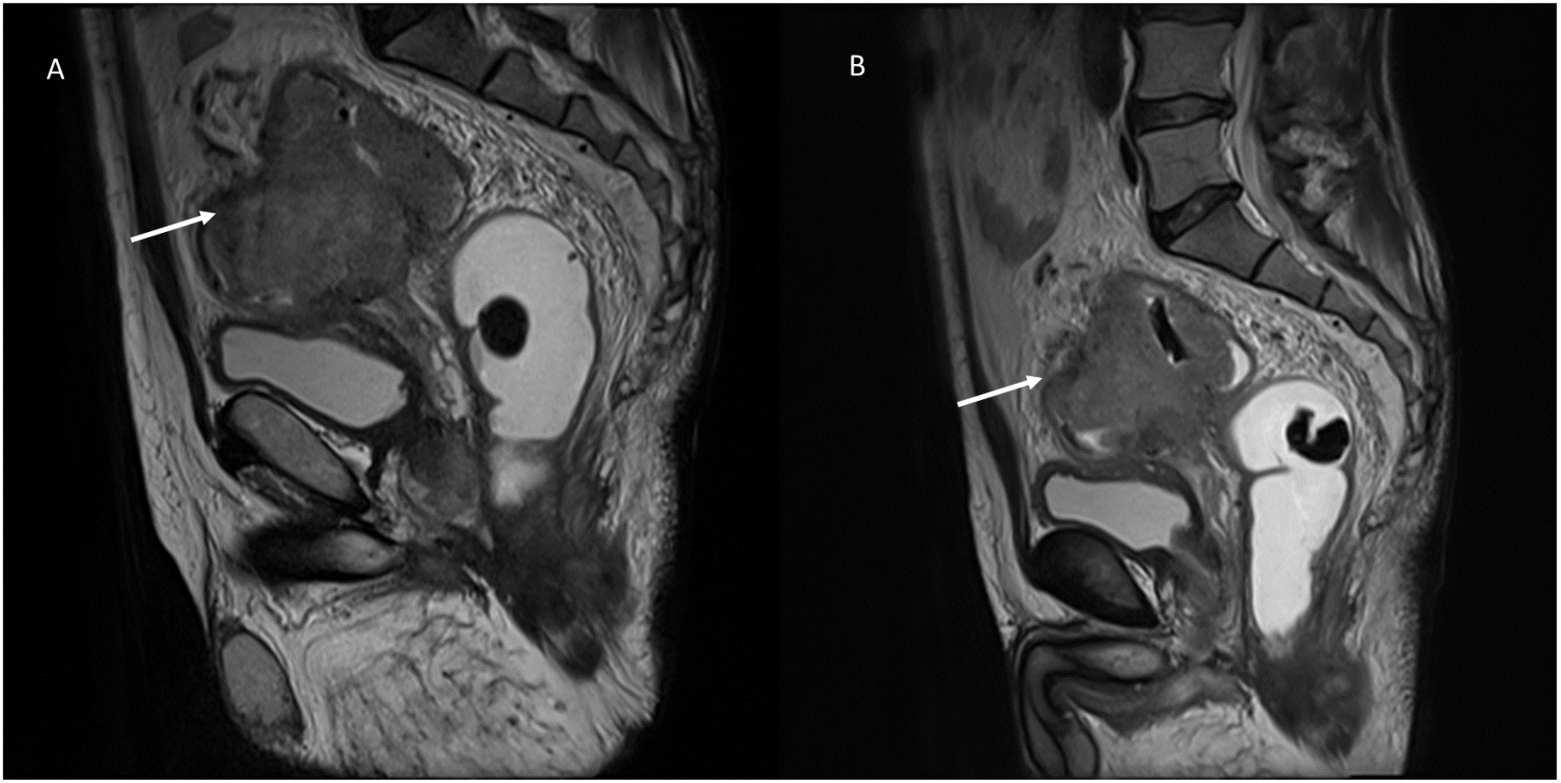
MRI evaluation of chemotherapy response in a patient with recto-sigmoid cancer. Sagittal T2-weighted MRI sequences of a patient, obtained in the pre-treatment **(A)** and post-treatment phase **(B)** after chemotherapy. **(A)** The pre-treatment image demonstrates a large, irregularly marginated mass (white arrow) with intermediate-to-hyperintense signal intensity relative to the surrounding soft tissues. The tumor partially encroaches on the adjacent structures. **(B)** The post-treatment image acquired after chemotherapy reveals a significant reduction in tumor volume (white arrow), indicating a favorable treatment response. Despite the size reduction, the lesion maintains similar T2-weighted signal intensity compared to the baseline examination. This figure highlights the importance of volumetric assessment in chemotherapy response evaluation, emphasizing that tumor shrinkage is a common indicator of therapeutic efficacy. From Vanzulli and Albano, (42).

Conventional chemotherapy is based on the inhibition of rapidly growing cell division, which is one of the characteristics of neoplastic cells. In addition, some cytotoxic chemotherapy drugs, including doxorubicin, mitoxantrone, and cyclophosphamide, can kill tumor cells via an immunogenic cell death pathway, which activates innate and adaptive antitumor immune responses and has the potential to greatly increase the efficacy of chemotherapy (43). Chemotherapy, defıned as a cytotoxic therapy, disrupts basic cellular processes such as proliferation, maintenance, metastasis, angiogenesis, and apoptosis in all cells, not just those with oncogenic drivers. Chemotherapy works because cancer cells have developed greater dependencies on these processes than normal cells. They may further have an im- paired ability to survive cytotoxic stress than normal cells as well. In truth, all chemotherapies are targeted agents, we just lack a clear understanding of their targets in normal and neoplastic cells. Therapeutic effects are responsible for a dimensional reduction of the lesion, until it disappears. So that during imaging evaluation, at CT or MRI imaging (**Figure 1**), the density or the signal of the lesion, in the different phase of contrast study or on the different sequences of the study protocol, will not be different from the “baseline” examination, showing only reduced size (44).

However, although the target lesion does not show structural changes, the liver could have different effects, as damage induced from chemotherapy treatment that should be evaluated during liver imaging since these could have an influence on patient outcome. The chemotherapy-related complications, steatosis, chemotherapy-associated steatohepatitis (CASH), and sinusoid obstructive syndrome (SOS) might damage the parenchyma, affecting the functionality and consequently the patient outcome (45). In fact, either CASH and SOS are correlated with an increase of morbidity after liver resection (45).

Chemotherapy related hepatitis can be categorized histologically in 3 groups: hepatocellular, cholestatic, or mixed. On imaging assessment, it is possible to identify several findings as perihepatic fluid, hepatomegaly, periportal edema and lymphadenopathy (45). The main typical feature is the gallbladder wall thickening or gallbladder fossa edema. On US evaluation, typical finding is a parenchymal echogenicity decreasing with an increase of the portal vein conspicuity. During CT or MRI, this appears as liver attenuation decreasing or diffuse hyperintensity on T2-weigthed (T2-W) sequences, with inhomogeneous parenchymal enhancement during the contrast studies. Severe cholestatic hepatitis appears on MR cholangiopancreatography (MRCP) sequences as a decreasing of the tertiary bile ducts number (45).

Chemotherapy-induced steato-hepatitis (named as CASH) affects hepatic regeneration and place patients at risk for post-surgical liver failure (45). In addition, liver steatosis decreases the difference in contrast between parenchyma and liver metastasis, influencing the post treatment assessment. On US, steatosis or steato-hepatitis causes a focal or diffuse improved liver echogenicity. However, a focal deposition of fat or a focal fat sparing may mimic a liver metastasis, that should be differentiate considering the location, the shape, and the absence of mass effect on vascular or biliary tree. MRI could be a problem solving, confirming the diagnosis since steatosis shows a signal loss on opposed-phase T1-W sequence, compared to in-phase sequence (45). On unenhanced CT, a reduced hepatic-to splenic attenuation ratio verifies the presence of fat, so as an increase of cranio-caudal liver diameter and an increase of caudate-to-right lobe ratio are typical steato-hepatitis findings (45).

Sinusoidal obstruction syndrome (SOS), also named veno-occlusive disease, is due to critical injury of the sinusoidal endothelial cells correlated to the fibrous substance accumulation within venule walls and sinusoids causing different histological changes from sinusoidal dilation to occlusion (45). Macroscopically, the involved parenchyma has a bluish-red marbled appearance and for this reason this condition is named also as “blue liver syndrome”. During Imaging assessment, different degrees of portal hypertension, hepatosplenomegaly, recanalization of paraumbilical vein, ascites, gallbladder wall thickening, and portal vein thrombosis can be found. During contrast studies, liver parenchyma can have a diffuse and inhomogeneous hypo-attenuation or intensity, mainly located at the peripheral area and right hepatic lobe. On hepatobiliary phase (HBP) MRI an inhomogeneous reticular pattern is found in the non-tumor parenchyma. In severe cases, it is possible to find regenerative hyperplasia nodules and peliotic changes (46).

### Target therapies

**Figure 2:**
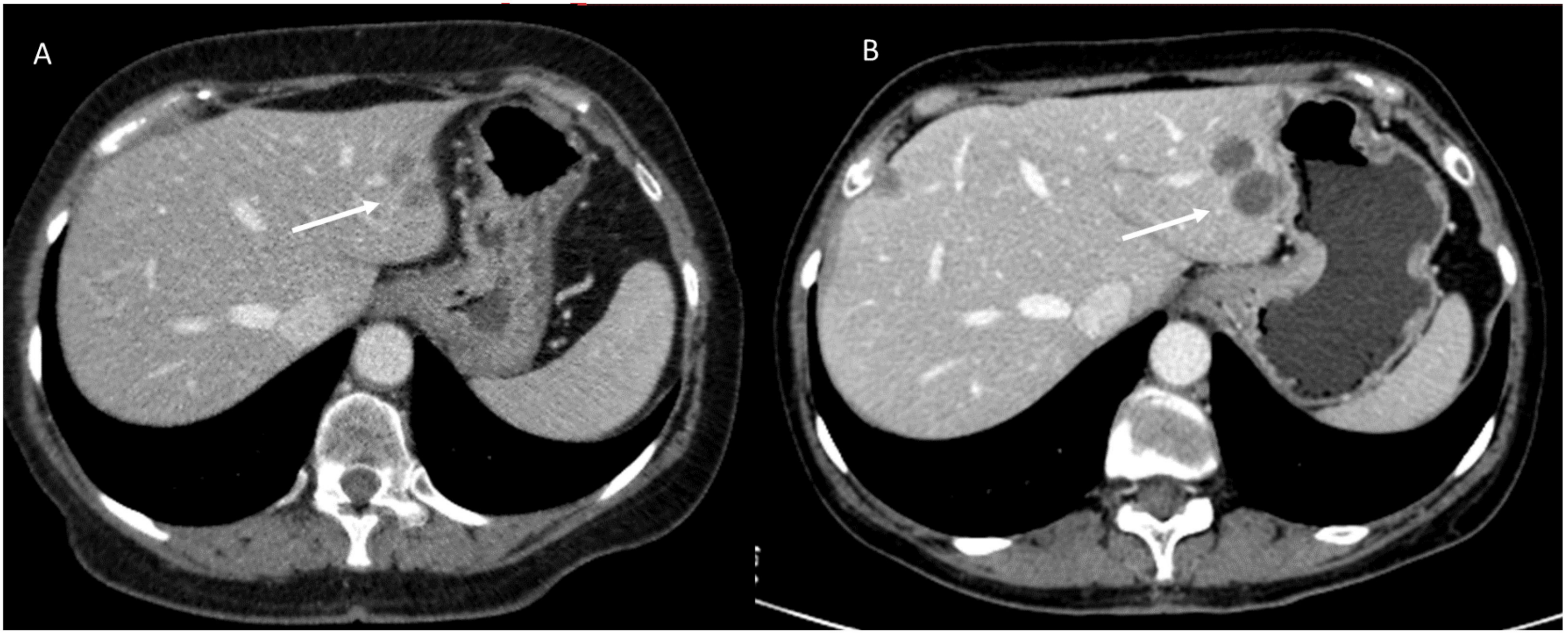
CT imaging assessment of target therapy response in a patient with colorectal liver metastases. Axial contrast-enhanced CT images in the portal venous phase: **(A)** (Pre-Treatment CT): A well-defined hepatic lesion (white arrow) is observed, exhibiting mild hyperattenuation in the portal venous phase and irregular borders. **(B)** (Post-Treatment CT): Follow-up imaging after targeted therapy demonstrates homogeneous hypoattenuation of the lesion (white arrow) with well-defined margins, consistent with a treatment response. However, there is an increase in lesion size. This imaging pattern underscores the importance of functional imaging to distinguish true disease progression from post-treatment changes. The increase in lesion size should not be mistaken for treatment failure without additional imaging or clinical correlation. From Vanzulli and Albano, (42).

Unlike standard chemotherapies, which inhibit normal cell division and kill rapidly dividing cells, including non-neoplastic cells, relatively indiscriminately, target therapies are designed to influence specific molecular signaling pathways important for the proliferation and survival of specific cancer cells (47). Target therapies are often classified by the nature of the agents: monoclonal antibodies (ending in “-mab”) show high specificity for antigens or receptors on the cell surface, and small molecule inhibitors (ending in “-ib “) able to cross the cell membrane and interact with intracellular targets. Tumor neoangiogenesis represents one of the major fields of application of target therapies (48). Angiogenesis is the process of vascularization of a tissue involving the development of new capillary blood vessels and is a highly controlled, physiological response that occurs mainly during the embryonic development, the female reproductive cycle and in the wound repair. Tumors, once they have reached a diameter of 2-3 mm, no longer being able to feed themselves by diffusion from the host’s microvasculature, must generate their own blood supply (49). This process is supported by the production of growth factors and proteolytic enzymes that stimulate tumor neoangiogenesis. Unlike physiological angiogenesis, tumor neoangiogenesis is characterized by chaotic, inefficient and permeable vessels. Antiangiogenic therapy reduces tumor perfusion, detectable on imaging by changes in attenuation and perfusion (reduction), while it has relatively limited effectiveness in reducing the size of a lesion because the mechanism of action of antiangiogenic agents is more cytostatic than cytotoxic (50). For example, liver metastases of colorectal cancer that respond to bevacizumab (**Figure 2**), an antiangiogenic monoclonal antibody, will show homogeneous hypoattenuation with well-defined margins, but may not decrease in size (51). Minor response and disease stabilization are observed in more than 70% of cases. Complete response to antiangiogenic therapy is rare, occurring in less than 1% of cases. Treatment resistance develops after 6-12 months of therapy (52). Fortunately, this resistance is specific to the antiangiogenic agent used and other antiangiogenic agents with different mechanisms of action can be used. Furthermore, after stopping the specific agent for a certain period (“drug holiday”), this specific resistance can be reversed (52). Following the resistance of the tumor to treatment, on imaging it is possible to observe the “rebound phenomenon”, which manifests itself as changes in the attenuation and perfusion (increase) of the lesion (51, 53). On MR imaging, the treated lesion, in response, in conventional sequences (T2-W and T1-W), will have a signal similar to the “baseline”. During contrast study, on CT than MRI, the lesion will show a less vascularity, with more defined margins (54). A semi-quantitative evaluation (with Dynamic Contrast Enhanced Magnetic Resonance Imaging-DCE-MRI) or quantitative (DCE-MRI and Diffusion Weighted Imaging - conventional DWI or Intravoxel Incoherent Motion Model (IVIM) and Diffusion Kurtosis Imaging - DKI) remains the more effective tool for treatment evaluation than the qualitative one, with intensity/time curves with reduced peaks and variations in quantitative parameters (e.g. Apparent Diffusion coefficient (ADC), pseudo-diffusivity (Dp), perfusion fraction (fp) and tissue diffusivity (Dt) (55–62).

Similar to conventional chemotherapy, target therapies can cause liver damage with radiological patterns similar to chemotherapy ones.

### Radiotherapy

**Figure 3:**
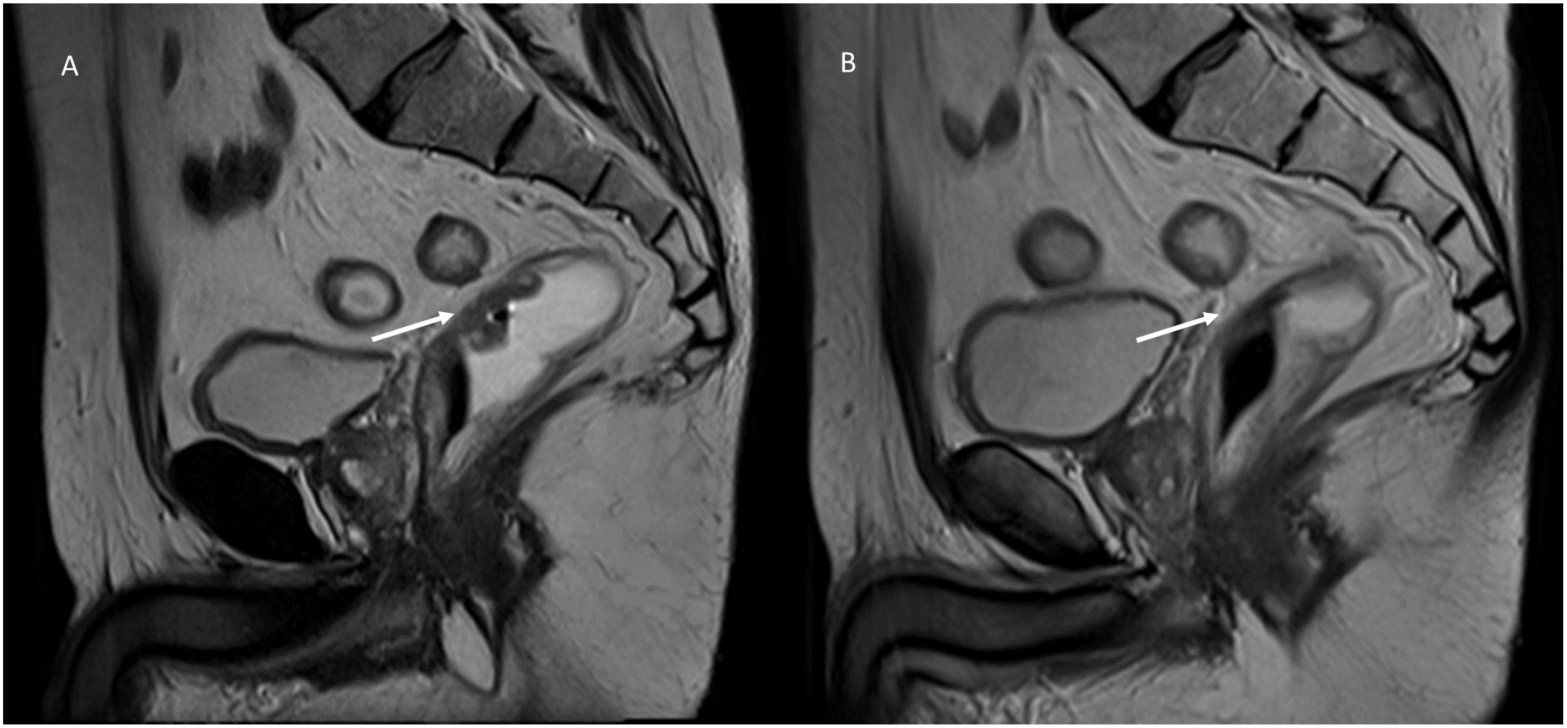
MRI assessment of post-radiotherapy fibrotic response in a patient with rectal cancer. Sagittal T2-weighted MRI images of a patient with T2-stage rectal cancer before and after short-course radiotherapy: **(A)** (Pre-Treatment MRI): The baseline T2-weighted sagittal image shows a well-defined, hyperintense rectal lesion (white arrow) in the mid-rectum; the lesion is infiltrating the rectal wall. **(B)** (Post-Treatment MRI): Follow-up MRI after short-course radiotherapy reveals a marked fibrotic response (white arrow). The lesion demonstrates a hypointense signal on T2-weighted imaging, with loss of the previously seen high signal intensity. The lesion’s borders have become less distinct, blending with the surrounding rectal wall, suggesting a treatment response with fibrosis replacing viable tumor tissue. From Vanzulli and Albano, (42).

Radiation is a physical agent, which is used to destroy cancer cells. The radiation used is called ionizing radiation because it forms ions (electrically charged particles) and deposits energy in the cells of the tissues it passes through. This deposited energy can kill tumor cells or cause genetic changes resulting in tumor cell death. High-energy radiation damages the genetic material (deoxyribonucleic acid, DNA) of cells, thus blocking their ability to divide and proliferate further (63–65). Although radiation damages both normal and cancer cells, the goal of radiation therapy is to maximize the radiation dose to the cancer cells while minimizing exposure to normal cells that are adjacent to the lesion or in the radiation pathway. Normal cells can usually repair themselves at a faster rate and maintain their normal state of functioning than cancer cells. Cancer cells in general are not as efficient as normal cells in repairing damage caused by radiation treatment. Radiation can be administered either with curative intent or as a palliative treatment to relieve patients of symptoms caused by cancer (66). Additional indications of radiation therapy include strategies for combination with other treatment modalities such as surgery, chemotherapy, or immunotherapy. If used before surgery (neoadjuvant therapy), radiation will aim to shrink the tumor. If used after surgery (adjuvant therapy), radiation will destroy microscopic tumor cells that may not have been eradicated (66). Tumors differ in their sensitivity to radiation treatment. There are two treatment modalities, with external or internal beam. In external beam treatment, radiation is delivered from outside the body by aiming high-energy beams (photons, protons, or particle radiation) at the tumor (67–70). This is the most common approach in clinical settings. Internal radiation or brachytherapy is delivered from inside the body by radioactive sources, sealed in catheters or seeds directly into the tumor site. It is used particularly in the routine treatment of gynecological and prostate cancers, as well as in situations where retreatment is indicated, based on its short-range effects (68–70). In evaluating treatment efficacy, both the effects on the target and on healthy neighboring tissues must be considered. In treated lesions, a reduction in size may be accompanied by the occurrence of fibrotic tissue (fibrotic response) (**Figure 3**), which must be properly identified, and characterized, for appropriate assessment of response (treatment efficacy) (71–76). On MRI imaging, a fibrotic tissue shows a hypointense signal on T2 weighted MRI sequences with a slow and progressive contrast take during contrast studies on CT than on MRI (77). In DWI, the signal may be restricted similar to pre-treatment, since fibrosis can cause restricted diffusion. In these cases quantitative evaluation with DCE-MRI and DWI or IVIM and DKI remains the more effective tool for treatment assessment compared to qualitative one, with intensity/time curves with reduced peaks and variations in quantitative parameters (ADC, Dp, fp and Dt) (77–83).

### Immunotherapy

**Figure 4:**
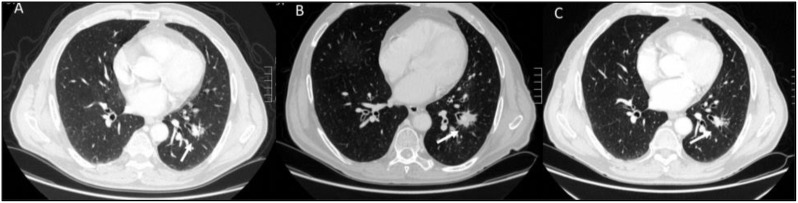
CT imaging evaluation of pseudoprogression and treatment response in a patient with lung cancer undergoing immunotherapy. Axial CT scans: **(A)** (Baseline CT, Pre-Treatment): The initial CT scan reveals a well-defined, solid lung mass in the left lower lobe. The lesion has irregular margins and is associated with some surrounding ground-glass opacities. **(B)** (First Evaluation, 3 Weeks After Immunotherapy): Follow-up imaging shows an apparent increase in lesion size (white arrow) with new areas of ground-glass opacity and perilesional infiltration. This pattern is consistent with pseudoprogression. **(C)** (Second Evaluation, 6 Weeks After Immunotherapy): A subsequent CT scan at 6 weeks demonstrates partial response to treatment, with a reduction in lesion size (white arrow). This confirms that the initial apparent progression was due to pseudoprogression rather than actual tumor growth. From Vanzulli and Albano, (42).

Immunotherapy denotes an exemplary change in cancer treatments. Indeed, compared to other therapies such as conventional chemotherapy, radiotherapy or targeted therapies, which target cancer, these treatments work by stimulating the patient’s immune system to achieve an immune reaction against cancer cells (84–87). Immunotherapy can be classified as passive or active, depending on the mechanism of action. In passive treatment, immunoglobulins can be administered and bind to tumor-related antigens; in active treatment, there is a stimulation of the immune system to target tumor antigens, thus, to have an effect against tumor cells. Although different approaches are currently used in clinical and preclinical settings, checkpoint inhibitors (ICIs): of cytotoxic T lymphocyte-associated protein 4 (CTLA-4), programmed cell death protein-1 (PD-1) or PD-L1: PD-1 ligand (PD-L1), are the most used ones (84–88). Immunotherapy is based on a complicated process, which includes several phases, during which there is a stimulation of the immune system. As a result, a number of immune cells adhere to the lesion or lesions, resulting in an increase in tumor size and/or the appearance of new lesions (16, 87). This event causes an atypical imaging response pattern called pseudoprogression (**Figure 4**). Pseudoprogression is an unusual event, occurring in 4-10% of melanoma patients treated with immunotherapy (16, 87). This phenomenon is challenging for radiologists because there is no clear feature at the imaging that can identify it versus true progression or hyperprogression.

**Figure 5:**
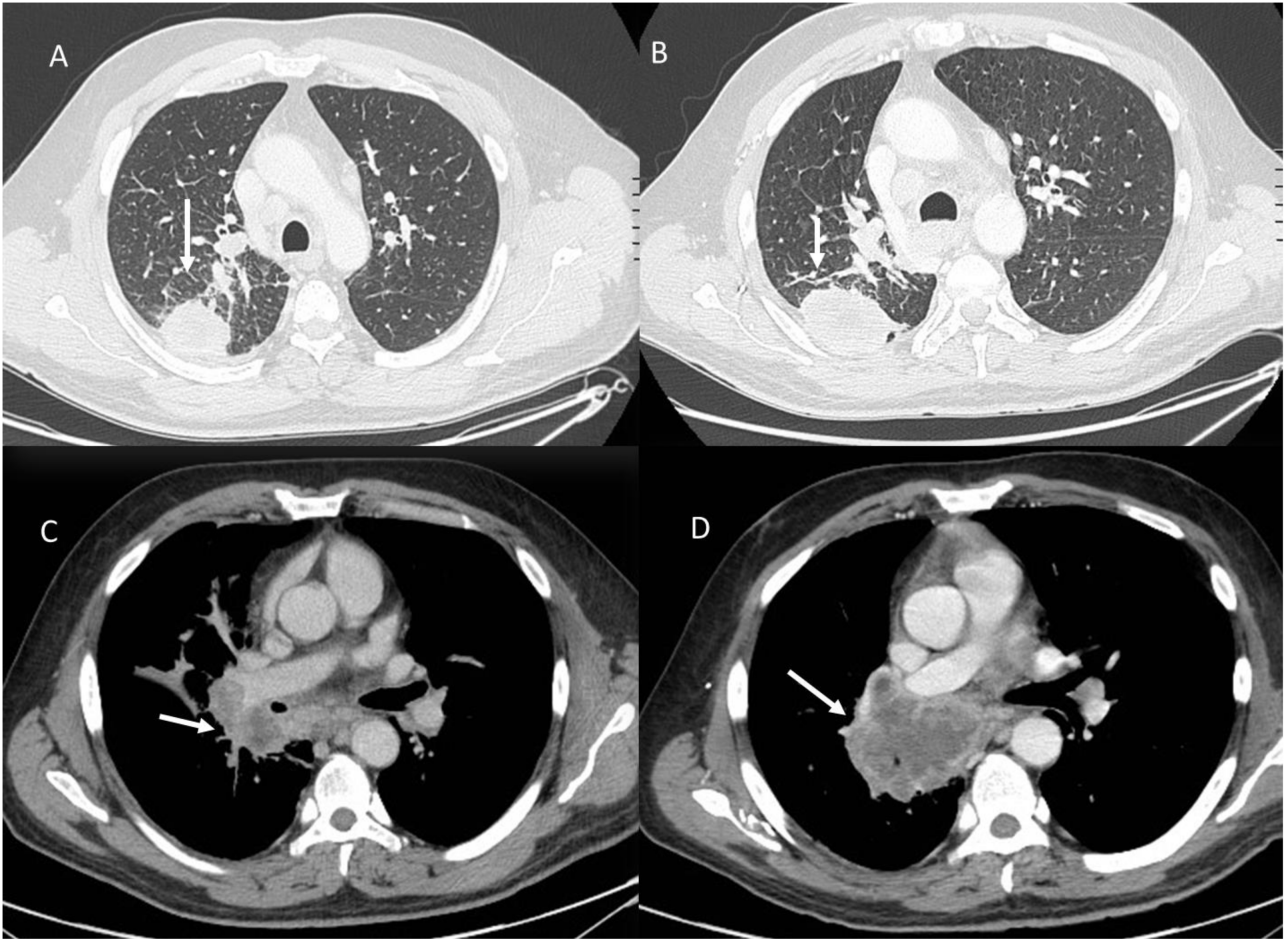
Hyperprogression during immunotherapy in a patient with lung cancer. Axial CT scans: **(A)** (Baseline CT with Lung Window): The initial CT scan shows a solid, spiculated mass in the left lower lobe (white arrow), with surrounding ground-glass opacities and an adjacent area of consolidation. **(B)** (Follow-up CT with Lung Window After Immunotherapy): Imaging performed during treatment evaluation reveals rapid tumor enlargement (white arrow) with new areas of consolidation and increased peritumoral opacities, suggesting disease progression. **(C)** (Baseline Contrast-Enhanced CT, Portal Venous Phase): Hilar metastasis (white arrow). **(D)** (Follow-up Contrast-Enhanced CT, Portal Venous Phase): Marked enlargement of the hilar metastasis (white arrow) with increased central necrosis is observed, further supporting hyperprogression. From Vanzulli and Albano, (42).

Hyperprogression (**Figure 5**) is a severe disease progression in which the growth rate of the lesion, after the initiation of treatment, increases by a factor of two.

**Figure 6:**
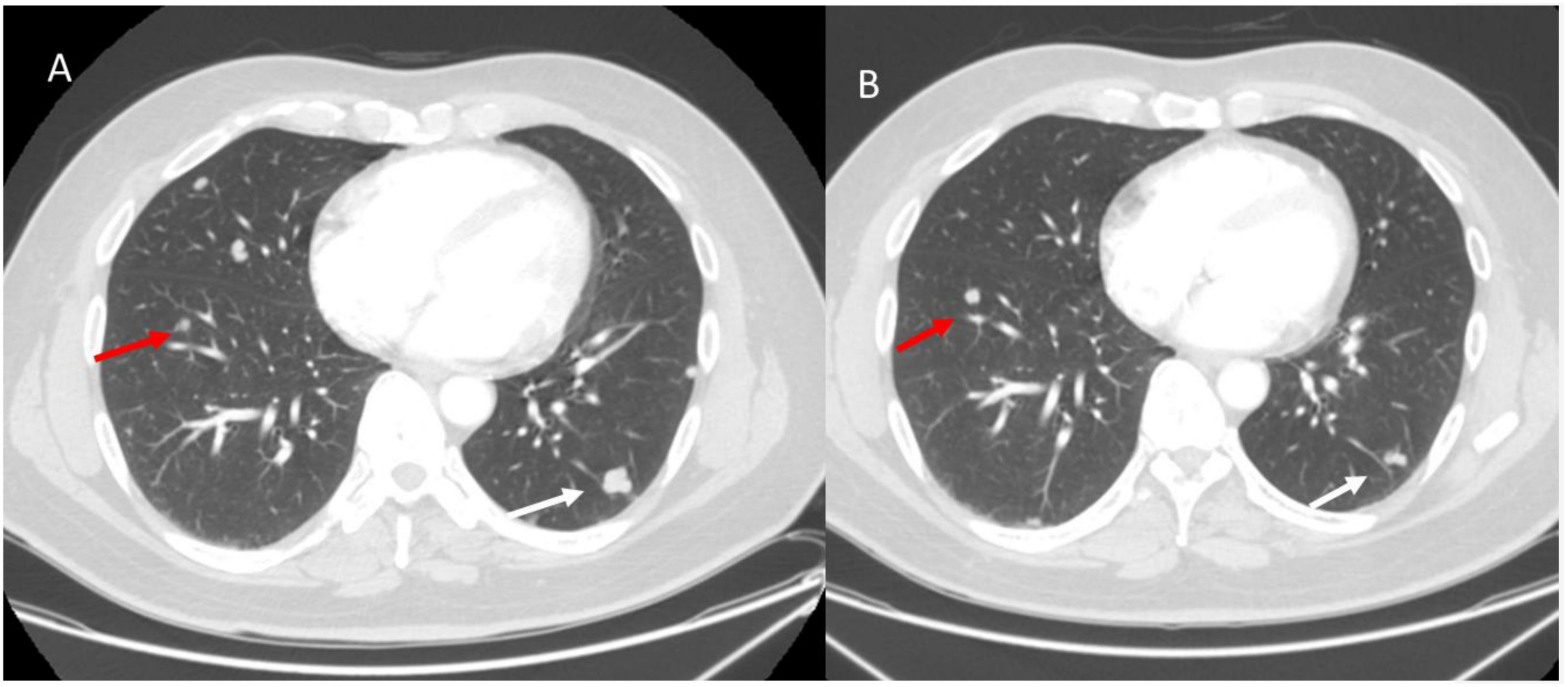
Dissociative response in a melanoma patient with lung metastases undergoing immunotherapy. Axial CT scans: **(A)** (Baseline CT, Pre-Treatment): Two metastatic lung lesions are identified: one in the left upper lobe (red arrow) and another in the right lower lobe (white arrow). Both lesions appear well-circumscribed with peripheral subpleural distribution. **(B)** (Follow-up CT After 3 Months of Immunotherapy): The right lower lobe lesion (white arrow) shows a significant reduction in size, indicating a partial response to immunotherapy. However, the left upper lobe lesion (red arrow) has increased in size, suggesting progressive disease in this specific lesion despite the overall tumor burden decrease. From Vanzulli and Albano, (42).

During immunotherapy, an additional atypical pattern is dissociated responses; this response is characterized by the presence of lesions that show a reduction in size, while others show an increase of size (**Figure 6**). This response pattern correlates with better survival than true progressions (16, 87).

### Ablation treatments and intra-arterial therapies

**Figure 7:**
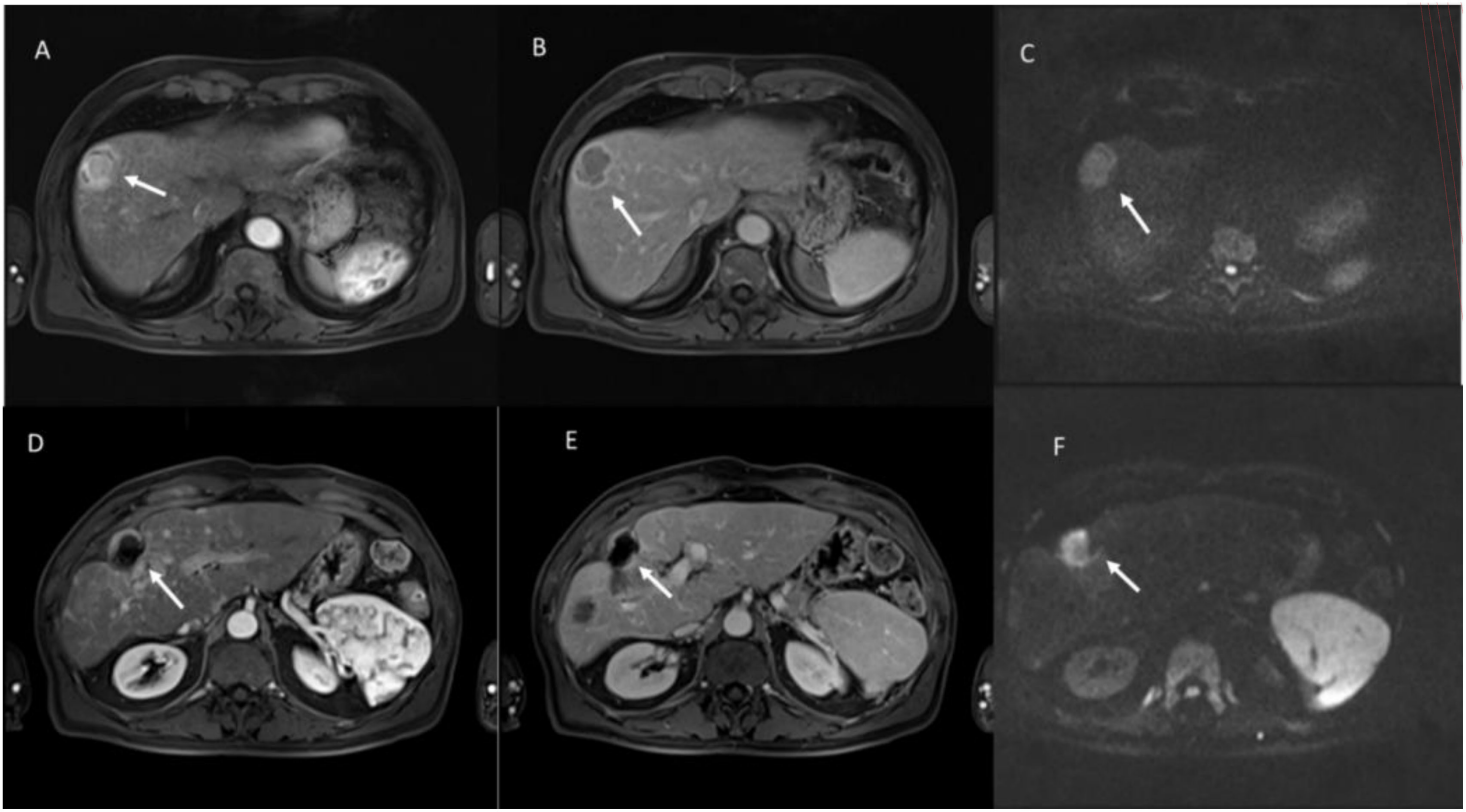
Multiparametric MRI assessment of evaluation of hepatocellular carcinoma (HCC) treated with radiofrequency ablation (RFA). Pre-Treatment Evaluation: **(A)** (Arterial Phase, T1-weighted VIBE FS): The lesion (white arrow) shows intense arterial hyperenhancement, a hallmark of viable HCC due to neovascularization. **(B)** (Portal Venous Phase, T1-weighted VIBE FS): The lesion exhibits washout, appearing hypointense compared to the surrounding liver parenchyma. **(C)** (Diffusion-Weighted Imaging, b800 s/mm²): The lesion demonstrates high signal intensity, indicating restricted diffusion due to high cellular density. Post-Treatment Evaluation (After RFA): **(D)** (Arterial Phase, T1-weighted VIBE FS): The lesion no longer exhibits arterial enhancement, with a hypointense center and peripheral rim, suggestive of post-ablation necrosis. **(E)** (Portal Venous Phase, T1-weighted VIBE FS): Persistent lack of contrast uptake confirms the presence of coagulative necrosis, indicating a successful ablative response. **(F)** (Diffusion-Weighted Imaging, b800 s/mm²): the treated lesion showed restricted diffusion for coagulative necrosis. From Vanzulli and Albano, (42).

Ablation treatments such as radiofrequency ablation (RFA) or microwave ablation (MWA), transcatheter arterial chemoembolization (TACE), and transcatheter arterial radioembolization (TARE) with yttrium 90 induce cell death or necrosis (89–93). These therapies can lead to tumor size stability or even increased tumor size after therapy, a feature that limits the role of size-based criteria. RF and MWA are the most used techniques. RFA produces necrosis due to thermocoagulation (89). With RFA, the active tissue-heating zone is restricted to a few millimeters near the electrode, while the target residue is heated by thermal conduction. Consequently, the effectiveness of the treatment is closely related to the size of the lesion and the maximum result is obtained for target lesions smaller than 3.5 cm. Furthermore, some tissue characteristics, such as electrical conductivity, thermal conductivity, dielectric permittivity, and blood perfusion rate, affect the effectiveness of the RFA procedure. RFA treatment should be avoided when the target is near large vessels because of the “heat sink effect” (89). MWA is based on the dielectric effect, which occurs when an imperfect dielectric material is subjected to an alternating electromagnetic (EM) field, generating a larger area of active heating (up to 2 cm near the antenna) allowing for necrosis more homogeneous in the target area, compared to RFA (89). Furthermore, MWA shows some improvements compared to RFA: the target can be larger in size as it generates a larger area of necrosis; treatment time is faster; the effectiveness is less influenced by the characteristics of nearby tissues, due to vaporization and carbonization, consequently, the heat sinking effect affects the effectiveness of MWA less (89). Both procedures are responsible for a coagulative necrosis (**Figure 7**), with modifications of the signal in conventional MRI sequences: in T1-W sequences the ablated area will have a hyperintense signal, while in T2-W sequences it is iso-hypointense, also in relation to the timing of the treatment (94, 95). During contrast studies, either on CT than on MRI assessment, a necrotic lesion appears devoid of vascularization, and the hyperintensity of the necrosis in T1-W sequences should not be considered as a non-responsive, for this reason a subtraction phase is advisable (96).

Treatments based on electroporation, namely electrochemotherapy (ECT) and irreversible electroporation (IRE), have recently emerged as possible alternatives to RFA and MWA, since they do not cause thermal necrosis but, by modifying the permeability of the cell membrane thanks to an induced electric field (electroporation), activate cellular apoptosis allowing better efficacy of a chemotherapeutic agent. The IRE is a direct ablation tool, as electroporation is used irreversibly. Several electrodes are placed around the target, using a series of high-voltage pulses of up to 3000 V and 50 A. These short-lived electric fields cause irreversible permeabilization of the lipid bilayer, disruption of cellular homeostasis, and stimulation of apoptotic pathways, causing the death of neoplastic cells. IRE does not cause damage to surrounding structures, such as vessels, and is preferred for lesions involving vascular structures. ECT is designed based on cell electroporation combined with the administration of a single dose of non-permeant or poorly permeant chemotherapeutic agents. The application of electric field to a cell causes a transient and reversible orientation of its polar membrane molecules, with an increase in permeability. This transient increase in permeability allows chemotherapeutic drugs to enter the cell, thus increasing the cytotoxic effects of the agents. This local enhancement allows to increase the effectiveness of chemotherapy on the target lesion, reducing the systemic effects of the drug, since it is administered at a low dose. On MR imaging three different layers can be identified in the area treated with IRE: an internal layer of coagulative necrosis (hyperintense in T1-W sequences and hypointense in T2-W sequences, without contrast uptake after its intravenous injection), an intermediate layer of congestion and hemorrhage (hypointense on T1-W, hyperintense on T2-W, with progressive contrast enhancement but with hypointense signal in the hepatobiliary phase), and a peripheral layer of inflammation (with hyperenhancement in the arterial phase but isointense in all other phases contrast and study sequences) (95–105). The size of the hepatobiliary phase ablation zone showed the highest correlation with the pathological size of the ablation zone (95).

Intra-arterial therapies such as transarterial embolization, TACE, and SIRT cause necrosis by selective, transarterial administration of different particles into the vessels supplying tumors (106, 107). Transarterial embolization involves the selective instillation of embolic materials (e.g. polyvinyl alcohol) into the hepatic arteries causing acute obstruction of the arteries feeding the lesion with subsequent exclusive ischemic necrosis (108). TACE involves the administration of chemotherapeutic agents (doxorubicin, cisplatin or mitomycin C) with or without the combination with embolic particles to increase the effectiveness of the treatment (109). SIRT, or radioembolization, is a form of brachytherapy that involves the intra-arterial administration of micron-sized particles (20-60 μm) containing yttrium 90 (90Y), which release focused β radiation to cause the destruction of the tumor while minimizing radiation damage to surrounding normal tissue (110). Compared to TACE, SIRT requires the preservation of adequate perfusion to the tumor to enhance free radical-dependent cell death from radiotherapy (110–115). In liver disease, in addition to their role in the treatment of locally advanced disease, intra-arterial therapies are increasingly used in addition to RFA to reduce the stage of tumors before surgical resection and as a bridge to liver transplantation. The lesion treated after intra-arterial therapies has a hyperintense signal in T1-W sequences and hypointense in T2-W sequences with lack of contrast uptake in sequences after intravenous administration of contrast medium. In the arterial phase, thin ring enhancement is possible (106). Issues inherent in the evaluation of response to treatment after TARE are related to the type of tumor, injection flow, timing of evaluation and number of embolization performed (106). Regarding the type of tumor, hypervascular lesions (HCC, neuroendocrine metastases) compared to hypovascular ones (colorectal liver metastases, cholangiocarcinoma) have different enhancement after treatment. Responding lesions will show reduction in size and decreased enhancement at 3 to 6 months (106). Many patients exhibit necrosis and/or peritumoral edema or inflammation, which may lead to an underestimation of response to treatment or a diagnosis of tumor progression. In some cases, a stable or even increased tumor size (pseudoprogression) is reported, with reduced blood supply to the tumor mass, probably due to tumor necrosis, hemorrhage or edema (106). Edema and inflammation appear as poorly defined geographic areas of low signal intensity on MRI during the portal/venous phase. This alteration can be easily characterized in DWI (no restriction) (106). Another common finding (in about a third of cases) is the presence, in the arterial phase, of a thin rim enhancement (usually less than 5 mm thick), which surrounds a treated lesion (106). The presence of peripheral nodules, evaluated as areas of enhancement, in growth represent a viable tumor residue, which may derive from an irregular distribution of the microspheres within the lesions. Many cases of nodular enhancement represent incompletely treated tumors that are located in the marginal area between two vascular distributions (106). Morphological changes in the liver may occur after TARE, especially in patients treated for liver metastases. These changes include atrophy of the treated lobe with contralateral lobar hypertrophy, increased splenic volume, and decreased diameter of the portal vein in the treated lobe may decrease, with an increase in the diameter of the contralateral intrahepatic portal vein and no change in the diameter of the splenic vein (106, 116–121).

### Integration of emerging techniques in radiomics and advanced quantitative imaging

The continuous evolution of imaging technologies has led to the development of more advanced techniques that complement radiomics in oncological imaging. Among these, artificial intelligence (AI), dual-energy computed tomography (DECT), and advanced quantitative imaging techniques such as perfusion MRI and diffusion-weighted imaging (DWI) have shown great promise in improving the non-invasive prediction of genetic mutations, treatment response, and overall prognosis in patients cancer disease (16, 53, 70, 80, 88, 92, 113).

In addition to traditional machine learning methods, deep learning (DL) approaches, particularly convolutional neural networks (CNNs), have demonstrated superior performance in medical imaging analysis. CNNs can automatically learn and extract complex image features from large datasets without requiring predefined radiomic feature extraction, potentially reducing human bias and improving predictive performance. Furthermore, deep learning-based radiomics (also known as deep radiomics) allows for end-to-end learning, enabling the integration of imaging features with clinical and molecular data to refine patient stratification. Unsupervised learning approaches, such as autoencoders and generative adversarial networks (GANs), can further enhance feature selection and optimize classification models, potentially surpassing conventional machine learning methods.

DECT is an advanced CT imaging modality that allows the differentiation of tissue composition based on the attenuation of X-rays at two different energy levels. This technique provides valuable quantitative information, including iodine uptake maps, virtual non-contrast images, and material decomposition analysis, which can enhance tumor characterization beyond conventional single-energy CT. Recent studies have suggested that DECT parameters, such as iodine concentration and spectral Hounsfield unit slope, may serve as imaging biomarkers for tumor aggressiveness, angiogenesis, and treatment response prediction. When integrated into radiomic workflows, DECT-derived features could complement traditional texture analysis. Additionally, DECT may aid in overcoming some limitations of standard CT-based radiomics by reducing beam-hardening artifacts and improving lesion conspicuity, particularly in cases with variable contrast enhancement (122, 123).

MRI-based quantitative imaging techniques, such as perfusion MRI and DWI, provide additional functional and microstructural information about tumor heterogeneity, which can further refine radiomics-based predictions. Perfusion MRI, through dynamic contrast-enhanced (DCE) imaging and dynamic susceptibility contrast (DSC) techniques, allows for the quantification of parameters such as blood volume, permeability, and perfusion kinetics. These biomarkers have been associated with tumor angiogenesis, a key factor in metastatic progression and response to anti-angiogenic therapies. By incorporating perfusion MRI-derived radiomic features, it may be possible to improve non-invasive predictions of molecular subtypes and treatment efficacy. Similarly, DWI has gained increasing attention in radiomics due to its ability to assess tissue cellularity and tumor microenvironment alterations. Apparent diffusion coefficient (ADC) values derived from DWI have been linked to tumor differentiation, hypoxia, and necrosis, all of which are relevant for assessing response to therapy and predicting genetic mutations. Advanced diffusion models, such as diffusion kurtosis imaging (DKI) and intravoxel incoherent motion (IVIM), provide even more detailed characterization of tumor microstructure and vascularity. Incorporating DWI-based radiomics into machine learning models could enhance the specificity of RAS mutation prediction and better guide therapeutic decision-making (77–83).

## Conclusion

In the era of personalized medicine, multiple therapeutic strategies are available for cancer management, often requiring a combination of treatments at different stages of disease progression. The radiologist plays a critical role in this process, not only in assessing treatment response but also in understanding the type, timing, and mechanism of action of the administered therapies. Effective imaging evaluation must consider not only the primary effects on the target lesion but also potential systemic and secondary effects, which may impact treatment planning and patient outcomes.

Beyond traditional imaging-based response assessment, radiomics and artificial intelligence (AI)-driven imaging analysis are emerging as transformative tools in oncologic decision-making. These advanced techniques have the potential to refine non-invasive characterization of tumors, predict treatment response, and personalize therapeutic strategies. However, several challenges remain in fully integrating imaging biomarkers into clinical practice. Standardization of imaging protocols, feature extraction methodologies, and AI-driven analytics is essential to ensure reproducibility and facilitate multi-center validation studies. Additionally, the integration of radiomics with other omics data (genomics, proteomics, and metabolomics) may further enhance predictive accuracy but requires interdisciplinary collaboration for proper interpretation and clinical translation.

Future research should focus on improving machine learning models by incorporating deep learning techniques, optimizing multi-parametric imaging approaches (including dual-energy CT and functional MRI), and establishing robust validation frameworks to confirm the clinical utility of radiomics-based predictions. Moreover, the development of explainable AI models is crucial to ensure that radiologists and oncologists can effectively interpret and apply imaging-derived insights in treatment decision-making.

Given the complexity of modern oncologic care, a strong interdisciplinary collaboration between radiologists, oncologists, medical physicists, and computational scientists is paramount. Radiologists must actively engage in treatment discussions, ensuring that imaging findings are contextualized within the therapeutic landscape. Standardized frameworks for treatment response assessment and complication classification must be adopted to ensure consistency across clinical settings.

In conclusion, the integration of advanced imaging techniques, radiomics, and AI into personalized cancer treatment holds great promise but requires addressing technical, methodological, and clinical challenges. A multidisciplinary approach will be fundamental in harnessing the full potential of imaging to optimize patient outcomes in the evolving landscape of precision oncology.
